# Toxicities of Therapeutic Agents Used in Medicine

**DOI:** 10.3390/toxics4030014

**Published:** 2016-07-27

**Authors:** Valentina Alda Carozzi, Guido Cavaletti

**Affiliations:** Experimental Neurology Unit, Neuroscience Center, School of Medicine and Surgery, University of Milan-Bicocca, Via Cadore 48, 20900 Monza, Italy; guido.cavaletti@unimib.it

This Special Issue on “Toxicities of Therapeutic Agents Used in Medicine” reports on some peculiar cases of toxicities related to widely and commonly employed drugs. Side effects are a universal problem that involve all medical disciplines, which doctors are forced to deal with in order to safeguard the effectiveness of therapies and at the same time the patients’ quality of life. Thanks to the progress of medical research, side effects can be often (but not always) managed. When the side effects of drugs are too debilitating, patients are forced to adjust or even suspend their therapies. Take for example the side effects of anticancer treatments: just a few years ago, side effects such as cardiotoxicity and myelotoxicity often forced the interruption of life-saving treatments. Now, thanks to the development of new less toxic drugs and some protective treatments, patients’ quality of life is more acceptable. 

In this Special Issue, most of the papers are focused on the adverse effects of agents occurring on the structure or function of the nervous system. The nervous system is particularly sensitive to toxic damage for several reasons. The first is that neurons are post-mitotic cells and once damaged, their repair is completely in the hands of the surviving cells. Recovery from severe neurotoxic cell death requires other neurons to expand their connections by axonal branching. Moreover, neurons are very long cells, vulnerable to attack at numerous sites: the cell body, dendrites, myelin, nodes, and synapses. Finally, while present, the blood brain barrier does not provide protection against lipid-soluble agents. Areas not completely protected by these barriers or areas where the barriers are fenestrated (sensitive ganglia), are particularly vulnerable and potential points of entry for toxic agents. Some neurotoxic agents can also exert their toxic effects on other body sites due to common damage pathways such as oxidative stress. This is one of the most studied mechanisms of drug toxicity and one of the keys events in the pathophysiology of peripheral neuropathy andof neurosensory hearing loss induced by platinum-based anticancer drugs. It is involved also in l-Dopa induced toxic effects on serotoninergic neurons in Parkinson’s disease and in the haemoglobin-based products induced disruption of renal as well as neuronal cells.

The highlights of this Special Issue can be summarized as follows:

1. Rentsendorj and colleagues reported a scientific paper that may have relevant implications for understanding the safety of haemoglobin-based products [1]. They examined in vivo the effects of the polymerized form of haemoglobin (HbG) on transcriptional regulation, activity, and expression of the renal antioxidant enzymes, to investigate its potential ability to promote oxidative tissue injury. Their findings provided evidence that renal exposure as well as central neurons exposure to HbG, (previously demonstrated), suppresses the function of the major antioxidant defence systems.
2. Chiorazzi and co-workers reviewed current views in platinum-related drug mechanisms that cause peripheral neurotoxicity. Cisplatin, carboplatin, and oxaliplatin, the three most famous neurotoxic platinum-based chemotherapy agents employed for the treatment of several solid tumours, affected the Dorsal Root Ganglia neuron machinery preferentially damaging mitochondria, membrane potentials, and anti-oxidative protective systems [2].
3. Callejo and colleagues examined in depth the state of art of cisplatin-induced ototoxicity. In addition to peripheral neurotoxicity, cisplatin produces a bilateral, progressive, irreversible neurosensory hearing loss due to the production of reactive oxygen species in the inner ear tissue. The authors reviewed the currently available preventive and protective strategies, discussing the problems related to the interfering effects of systemic administration and consequently promoting local injection strategies [3].
4. Bernocchi and coworkers studied the effects of cisplatin on the immature brain, which appears to be more vulnerable to injury than the adult brain. Changes in the intracellular calcium homeostasis within the central nervous system architecture after cisplatin exposure demonstrates that the equilibrium and synergy between calcium proteins to limit neuroarchitecture damages [4] is essential.
5. Argyriou discussed the important open issue of the availability of reliable biomarkers to allow prompt identification of patients at high risk of developing oxaliplatin-induced peripheral neuropathy. This review described the relationship between some peculiar genetic variants and the pathogenesis, clinical outcome, and management of peripheral neuropathy [5].
6. As reported by Nicolini and colleagues, not only the Dorsal Root Ganglia sensory neurons, but also axonal transport can be perturbed by antineoplastic agents. Axonal bidirectional trafficking along peripheral nerves can be impaired by both “old” but widely employed, and by “young” but less studied, chemotherapy agents [6].
7. Meregalli described some of the mechanisms related to bortezomib-induced peripheral neuropathy. Bortezomib is a proteasome inhibitor chemotherapy drug that was also recently considered to able to dysregulate tubulin disassembly and consequently alter axonal transport. However, other actors seem to also be involved in this story [7].
8. Other widely studied antitubulinic chemotherapy drugs are taxanes. Velasco and Bruna reviewed some of the most updated knowledge on the real incidence, pathophysiology, clinical features, and predisposing factors related to the development of taxane-induced peripheral neuropathy [8].
9. Not only taxanes, but also platinum compounds, vinka alkaloids, and proteasome inhibitors can induce a mitochondrial dysregulation in peripheral nervous systems during chemotherapy. Canta and collaborators reported that the dysfunction of calcium signalling pathways and the production of reactive oxygen species could determine abnormal membrane potentials and neuronal excitability. Genetic changes in mitochondrial DNA also lead to gradual neuronal energy failure [9].
10. Last, but not least, Stansley and Yamamoto reported the latest findings on the safety of l-Dopa for the treatment of Parkinson’s Disease. Since dopamine is produced by l-dopa in part by serotonin neurons, an increase in dopamine seems to cause oxidative stress and damage serotonin neurons. l-dopa also caused deficits in serotonin neurotransmission controlling mood and cognition, warranting some severe side effects observed in Parkinsons’ patients [10].

Taken together, these ten papers suggested that it is worth studying the adverse effects of a therapeutic drug as much as the beneficial effects, because in some cases their toxicity profile relies on their activity properties.
